# A Behavioral Ground Truth for Exteroceptive Sensors: Geometric Constraints and Stochastic Duration in Parking Maneuvers

**DOI:** 10.3390/s26061911

**Published:** 2026-03-18

**Authors:** Salvatore Leonardi, Natalia Distefano

**Affiliations:** Department of Civil Engineering and Architecture (DICAR), University of Catania, Via Santa Sofia 64, 95123 Catania, Italy; natalia.distefano@unict.it

**Keywords:** parking maneuvers, human baseline, geometric constraints, sensor calibration, ground truth, generalized linear models

## Abstract

**Highlights:**

**What are the main findings?**

- Aisle width in parking maneuvers generates distinct non-linear kinematic signatures, offering essential behavioral patterns for onboard sensor training.
- Furthermore, the temporal asymmetry observed between entering and exiting the parking lot defines dynamic tolerances, providing key parameters for obstacle tracking by autonomous sensors.

**What are the implications of the main findings?**

- The empirical ground truth dataset on parking dynamics enables the fine-tuning of exteroceptive sensors, preventing human hesitations from being classified as static obstacles.
- Integrating these metrics optimizes predictive logic, improving the reliability of sensory detection for autonomous parking systems in shared urban spaces.

**Abstract:**

The deterministic simplification of parking maneuvers in traditional traffic models presents a critical challenge for the safe integration of Autonomous Vehicles (AVs). This study establishes a stochastic human baseline to provide a naturalistic ground truth dataset essential for calibrating perception and prediction sensors in mixed traffic scenarios. Through the analysis of 1038 maneuvers observed in a university shared space in Catania, Generalized Linear Models and Kaplan–Meier estimators were applied to quantify the impact of geometric constraints on 0°, 45°, and 90° configurations. Results identify 45° angled parking as the Pareto-optimal solution regarding stability and speed, achieving an average maneuver time of 7.54 s. Furthermore, a vertical parking paradox emerges: in the presence of narrow aisles, entry times increase drastically, generating bottlenecks with an 85th percentile exceeding 50 s. Finally, a structural functional asymmetry reveals that exit maneuvers require approximately 54% of the time needed for entry. These findings provide empirical metrics essential for validating human behavior models and fine-tuning decision-making and timeout logic in autonomous driving systems.

## 1. Introduction

Parking management constitutes one of the most complex, pervasive, and strategically relevant issues in contemporary sustainable urban mobility planning. It acts not merely as a static vehicle storage service, but as a critical dynamic interface mediating the complex, bidirectional interaction between the demand for punctual accessibility to various urban functions (residential, commercial, work, recreational) and the residual capacity of the local road network, directly influencing congestion levels, energy efficiency, and urban quality of life [1]. Although consolidated technical literature and international regulatory frameworks [2,3,4] have extensively codified the geometric and dimensional criteria for parking area design—focusing predominantly on the macroscopic Supply Demand Balance and the optimization of surface utilization rates—the microscopic, behavioral, and dynamic component of the phenomenon has historically been treated with less analytical rigor and often relegated to a secondary role in transport modeling [5].

Specifically, the time interval required to execute entry and exit maneuvers, which constitutes the actual operational impedance imposed on the circulating flow, is often overlooked or overly simplified in traditional traffic assignment models [6]. Fundamental professional reference manuals, such as the ITE Parking Generation Manual or the Highway Capacity Manual [7], systematically tend to reduce maneuver time to a deterministic constant or a flat average value (e.g., a fixed time per maneuver regardless of the geometric or environmental context), failing to capture the intrinsic variance, heteroscedasticity, and “long tails” of the temporal distribution linked to the human factor (age, experience, psychophysical state, gender) and micro-geometric environmental conditions (lighting, weather, visibility, pavement status). This deterministic simplification proves particularly inadequate and potentially misleading in reflecting the stochastic, non-linear, and often unpredictable nature of real driving behavior [8], leading to systematic errors and significant underestimates in evaluating infrastructure operational capacity and Levels of Service (LOS). This discrepancy between theoretical models and operational reality becomes critical, especially in complex environments defined as “Shared Spaces” [9], where the functional distinction between vehicular and pedestrian spaces is blurred or absent.

In contexts such as university campuses, intermodal areas, historic centers, or large commercial parking facilities, characterized by mixed traffic and flexible regulation, interactions between users cease to be regulated by rigid rules and become continuous, implicit social negotiations based on non-verbal language (e.g., eye contact, vehicle position, gestures) [10]. In these high-interaction density scenarios, trajectory ambiguity and severe spatial constraints transform every single maneuver from an isolated event into a potential local disturbance event [11]. Such an event is capable of triggering hysteresis phenomena in vehicular outflow, propagating stop-and-go waves and generating non-linear upstream congestion that can paralyze entire sectors of the adjacent road network, with negative repercussions on the system’s overall safety and efficiency [12,13].

The urgency for more accurate, granular, and data-driven parameterization is exponentially amplified today by the ongoing transition towards connected, cooperative mobility and Intelligent Transport Systems (ITS). Current Path Planning algorithms, trajectory control, and decision-making of Autonomous Vehicles (AVs) require extremely robust, reliable, and realistic predictive models to interact safely and efficiently with human drivers in mixed traffic scenarios, which will characterize mobility in the coming decades [14,15]. The most recent literature in the field of Human–Robot Interaction strongly emphasizes how AVs, to be effectively integrated and socially accepted into the urban fabric, must adopt driving styles that are not only technically safe but “socially acceptable” (Human-Like Driving) [16,17]. Indeed, the social acceptance of these technologies strictly depends on the users’ trust in automated systems, a dynamic recently demonstrated in studies regarding the perception of Advanced Driver Assistance Systems (ADAS) in southern Italy [18]. This implies the autonomous system’s ability to understand, predict, and, if necessary, emulate the timing, hesitations, and execution modes of human maneuvers to avoid conflicts, misunderstandings, or gridlock situations that could compromise traffic fluidity. In the absence of a reliable “Human Baseline,” i.e., kinematic “Ground Truth” data describing the real variance of lane occupation times, the calibration of exteroceptive sensors (such as LiDAR, radar, and vision cameras for environmental perception) risks being flawed. Automated systems, lacking validated empirical references, risk adopting sub-optimal behaviors: on the one hand, overly conservative algorithms could impose disproportionate safety margins [19]; on the other, the failure to parameterize “human hesitations” could lead the system to erroneously classify a complex maneuver as a sensor anomaly [20]. In particular, the estimation of critical Gap Acceptance and clearance times during complex and delicate perpendicular or parallel parking operations remains one of the most significant and difficult open challenges for the validation and certification of SAE Level 4 Automated Valet Parking (AVP) systems, where the vehicle operates autonomously within dedicated or mixed infrastructures without direct human supervision [21,22].

Furthermore, while numerous simulation and theoretical studies have focused on the ideal kinematics of maneuvers in open spaces [23,24], there is a surprising lack of works that have empirically investigated the physical interaction between parking lot geometry and lateral spatial constraints (aisle width) in naturalistic and uncontrolled contexts [25,26]. This knowledge gap is critical, as engineering evidence and practical experience suggest that aisle width is a determining factor, often even more so than the parking lot angle itself, for the kinematic feasibility of direct entry and the cognitive load imposed on the driver [27]. Bridging this fundamental knowledge gap, the present study proposes a rigorous quantitative analysis based on a naturalistic dataset of 1038 maneuvers observed at the University Campus of Catania, an environment that constitutes an ideal open-air laboratory due to its morphological and functional heterogeneity. Unlike classical approaches, which are often limited to static comparisons between standard geometries (0°, 45°, 90°) under ideal conditions [28], this work adopts an explicitly data-driven perspective to investigate the critical, non-linear, and often counter-intuitive interaction between geometric configuration and available maneuvering space.

It is fundamental to highlight that the objective of this work is not to simulate the optimized execution times of a fully autonomous vehicle, where the variance of human skills would naturally be eliminated by sensor precision. Instead, this study aims to establish a reliable “Human Baseline” designed specifically for mixed traffic scenarios. Since autonomous vehicles will share urban spaces with human drivers, the variance inherent in manual driving is not a statistical noise to be removed, but the essential behavioral “Ground Truth” that predictive algorithms must learn to interpret to avoid classifying physiological hesitations as anomalies.

The ultimate goal is to provide empirical impedance functions, probability curves, and essential operational parameters, establishing an indispensable “Ground Truth” reference for the development of more efficient, adaptive, and “human-aware” AV control logic, thereby contributing to the creation of safer, more resilient, and higher-performing future transport systems.

## 2. Materials and Methods

The methodology adopted in this study has been rigorously structured to ensure the logical consistency, scientific replicability, and inferential robustness of the analysis, following a sequential workflow that proceeds from the characterization of the physical environment to the advanced mathematical modeling of the observed phenomena.

The section is therefore organized into three distinct and interdependent subsections, each dedicated to a specific phase of the research process.

First, the study context and the engineering and operational criteria guiding the selection of survey sites are detailed, describing the geometric and functional features of the monitored scenarios to represent a comprehensive spectrum of urban parking conditions.

Subsequently, the experimental data acquisition protocol implemented in the field is illustrated, specifying the technological instrumentation used for high-precision temporal measurement, the kinematic definitions adopted for maneuver segmentation, and the taxonomy used for the classification of explanatory variables related to vehicles and drivers.

Finally, the analytical framework defining the statistical architecture of the study is presented, justifying the choice of the implemented inferential models in relation to the stochastic and non-normal nature of the collected data, and outlining the logical path leading from raw data to the estimation of impedance functions.

### 2.1. Study Context and Site Selection

The experimental investigation was conducted within the University Campus of Catania, an area that serves as an open-air laboratory representative of modern urban “Shared Spaces”. Building upon previous investigations that highlighted its complex accessibility dynamics [29], the site was specifically selected to monitor naturalistic parking maneuvers under mixed and unregulated traffic conditions, characterized by the continuous coexistence of vehicular, pedestrian, and cyclist flows.

**Figure 1:**
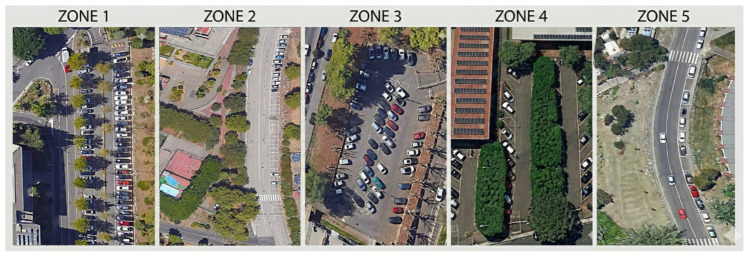
Aerial view of the five monitored parking zones.

To isolate the influence of geometric variables on maneuver duration, five distinct parking areas were selected, covering the full spectrum of standard configurations, with specific attention to the variability of available maneuvering space (Figure 1). To ensure geometric consistency across the analysis, it is essential to note that all the parking lots monitored in the different campus zones present uniform standard dimensions, specifically 2.50 m in width and 5.00 m in length. These measurements strictly comply with the Italian national regulatory standards for parking facilities. Since the internal dimensions of the individual parking areas remain constant across all the investigated configurations (0°, 45°, and 90°), this geometric parameter does not introduce any variance into the dataset. Consequently, the structural variance in maneuver times is exclusively driven by the interaction between the parking angle and the available lateral space, namely the aisle width.

The methodological core of the research lies in the sampling strategy adopted for perpendicular parking lots (90°), deliberately selected in two antithetical spatial configurations to evaluate the impact of lateral constraints.

The first scenario, identified at the Department of Engineering parking area (Zone 1), represents a condition of severe geometric constraint: the area features orthogonal parking lots served by two one-way maneuvering aisles only 3.00 m wide, delimited by physical curbs and trees. Although critical compared to regulatory standards, this configuration was included in the study to analyze user behavior under “geometric deficit” conditions, simulating high-density scenarios typical of historic centers or dated underground structures. In contrast, the second perpendicular scenario was located along the access road to the CUS (University Sports Center), where parking lots face directly onto the roadway (Zone 2). In this “unconstrained” context, the driver benefits from virtually unlimited maneuvering space, being able to utilize the opposite lane for setting the entry trajectory.

To complete the experimental framework, angled parking configurations (45°) were monitored at the Departments of Mathematics and Chemical Sciences (Zone 3 and Zone 4). The decision to monitor two distinct zones for the 45° parking geometry was primarily driven by the necessity to assemble a robust and statistically significant sample size. Since the individual parking areas at the University Campus feature a limited number of angled lots, a single zone could not provide the required daily turnover to achieve a high volume of observations within the strict temporal filtering applied to our survey. By combining data from the adjacent Departments of Mathematics (Zone 3) and Chemical Sciences (Zone 4), which share identical geometric and functional characteristics for the 45° configuration, we successfully gathered a comprehensive dataset. This combined approach captures a wider naturalistic variance in driver behavior, thereby strengthening the reliability of the derived human baseline.

Finally, the parallel parking configuration situated along the perimeter road (Zone 5) was monitored, thus allowing for a transversal comparison across all urban parking typologies.

### 2.2. Data Acquisition Protocol and Instrumentation

**Figure 2:**
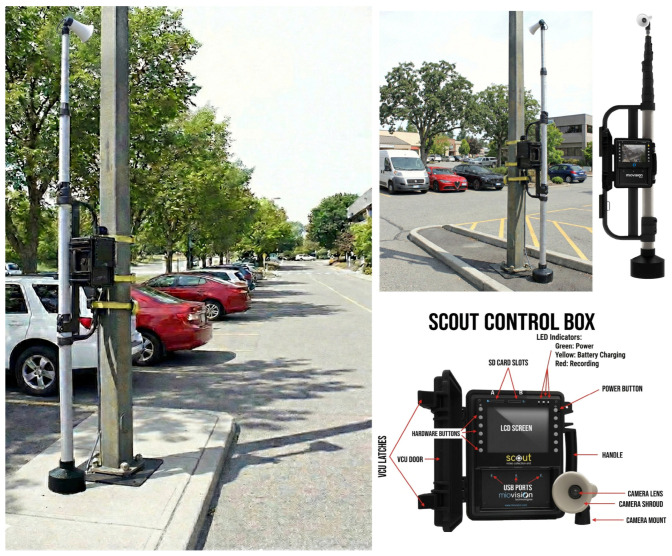
The MioVision Scout equipment and examples of deployment locations used for the field survey.

The data collection campaign was conducted using a non-intrusive naturalistic approach, leveraging the Department’s instrumentation, which includes two twin MioVision Scout VCU (Video Collection Unit) automated acquisition systems (MioVision Technologies Inc., Kitchener, ON, Canada) (Figure 2).

The simultaneous deployment of these two units, both equipped with an integrated telescopic mast (Power/Mast configuration) extendable to an operating height of approximately 6 m, allowed for the optimization of the experimental campaign schedule by parallelizing monitoring sessions across different sites, while ensuring a quasi-orthogonal aerial perspective of the road surface.

This hardware configuration minimized dynamic visual occlusions between vehicles and eliminated parallax error, thereby preserving the spontaneity of driving behavior due to the absence of visible operators at the roadside. Data reliability under variable environmental conditions was ensured by the system’s rugged specifications, featuring IP65 weather resistance certification and extended power autonomy (up to 7 days with an external battery pack), which enabled continuous monitoring without operational interruptions.

To ensure a representative and unbiased dataset, the video recording campaigns were systematically conducted in the year 2024 during the academic semesters, exclusively on weekdays from Monday to Friday. The recording windows were strategically concentrated during the peak and sub-peak operational phases of the University Campus, specifically between 08:00 a.m. and 02:00 p.m., to capture the naturalistic physiological flow of arrivals and departures. Furthermore, weekends, national holidays, and days characterized by anomalous events, such as graduation sessions or massive student assemblies, were deliberately excluded from the survey. This strict temporal filtering was implemented to prevent unusual behavioral patterns, extreme congestion levels, or entirely empty parking areas from skewing the naturalistic human baseline measured in the field.

The kinematic data extraction process was performed during the offline video post-processing phase, replacing manual stopwatch measurement with a rigorous frame-by-frame analysis. Maneuver duration (T_man_) was determined with decimal temporal resolution by identifying the key-frames corresponding to critical timestamps:

- t_0_: instant of longitudinal speed cancellation (setup stop);
- t_end_: instant of complete static alignment within the parking lot (for entry) or recovery of operating speed within the traffic flow (for exit).

Concurrently with the kinematic analysis, metadata regarding vehicle classification and boundary conditions were extracted. The system allowed for vehicle categorization according to three reference dimensional macro-classes: Small (City cars and Subcompacts, e.g., EU Segments A/B), Medium (Compact cars, e.g., EU Segment C), and Large (Mid-size/Large sedans and SUVs). Other contextual variables, such as adjacent parking lot occupancy, were also recorded.

It is essential to highlight that this sample was not generated by a predefined focus group or a restricted set of individuals. Due to the continuous and naturalistic monitoring of the diverse user population navigating the campus in 2024, the measurement process was completely random and observational. This ensures that the dataset intrinsically captures a vast plurality of different drivers and vehicles, thereby minimizing the risk of repeated measures from the same subjects biasing the results and providing a genuine representation of physiological driving variance.

The resulting dataset, validated and cleansed of anomalous events, consists of *N* = 1038 valid observations, distributed among the monitored configurations as follows:

- Vertical parking (90°): 259 maneuvers at the restricted site (Zone 1) and 229 at the unrestricted roadside site (Zone 2);
- Angled parking (45°): 322 total observations, split between the Zone 3 (213) and the Zone 4 (109);
- Parallel parking: 228 maneuvers recorded along the perimeter road axis (Zone 5).

### 2.3. Analytical Framework

The preliminary statistical analysis conducted on the overall dataset revealed a complex data structure characterized by marked positive skewness. Specifically, maneuvers performed at the restricted site (Zone 1) exhibit a “heavy tail” of extreme values, attributable to the multiple corrective iterations imposed by the undersized aisle width (W_A_ = 3.00 m). To formally assess these distributional properties, the Shapiro–Wilk test was applied, strictly rejecting the null hypothesis of normality for maneuver times across all geometric configurations. Concurrently, the Breusch–Pagan test confirmed the presence of significant heteroscedasticity, providing statistical evidence that the variance of execution times increases systematically with the geometric difficulty of the parking layout. Consequently, the application of standard techniques based on the Normal distribution (e.g., ANOVA or simple linear regression) would be biased and inadequate for handling the heteroscedasticity (non-constant variance) intrinsic to the phenomenon. Indeed, as maneuver difficulty increases, not only does the mean time grow, but the performance variability among different drivers also increases exponentially.

**Figure 3:**
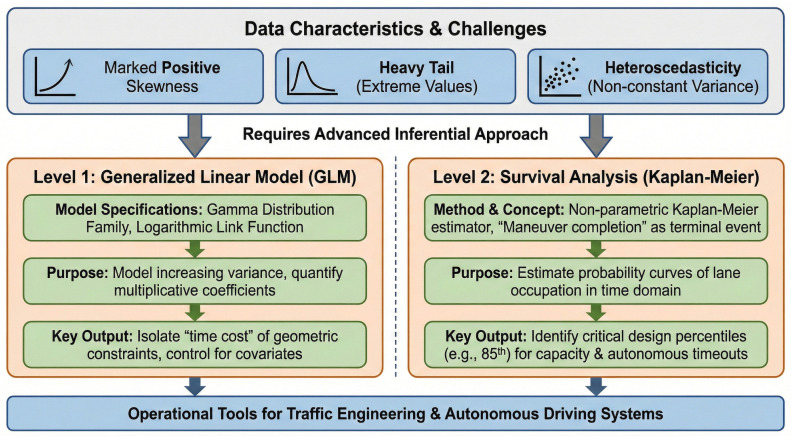
Schematic representation of the adopted analytical framework.

Therefore, the analytical framework was structured into two advanced inferential levels specifically designed for positive temporal variables (Figure 3).

First, a Generalized Linear Model (GLM) was developed by specifying a Gamma distribution family with a logarithmic link function. This mathematical configuration was selected for its ability to model increasing variance, allowing for the precise quantification of multiplicative coefficients associated with different factors. This approach isolated the pure “time cost” of the geometric constraint (the differential between off-street and on-street sites) while controlling for demographic covariates (age, gender) and vehicle type.

In parallel, to translate these results into operational tools for traffic engineering, Survival Analysis was applied using the non-parametric Kaplan–Meier estimator. By treating “maneuver completion” as the terminal event, the model was configured to estimate the probability curves of lane occupation in the time domain. These curves provide a direct and dynamic measure of the impedance imposed by each geometry, allowing for the identification of critical design percentiles (e.g., the 85th percentile) necessary for calculating residual capacity and defining timeout thresholds in autonomous driving algorithms.

Within this analytical framework, the operational definition of “Ground Truth” adopted in this study focuses on macroscopic and temporal metrics rather than low-level continuous kinematic parameters, such as high-frequency vehicle trajectories, speeds, accelerations, or steering angles. While these high-frequency measurements remain crucial for the internal proprioceptive tracking and the physical control of the autonomous vehicle, the macroscopic metrics derived here are specifically tailored to calibrate exteroceptive perception systems. For autonomous systems navigating mixed traffic, establishing a “Behavioral Ground Truth” based on the statistical awareness of lane occupation time is essential to effectively differentiate between physiological human hesitations and actual static anomalies, thereby optimizing the predictive logic of external tracking sensors.

Furthermore, while the preliminary Generalized Linear Model (GLM) initially controlled for demographic covariates (age, gender) and vehicle type, a stepwise model selection process revealed that their statistical significance was negligible compared to the explanatory power of the geometric layout and lateral spatial constraints. Consequently, to ensure statistical parsimony, these variables were excluded from the final model specification, confirming that severe spatial restrictions structurally dominate individual subjective differences or vehicle dimensions.

## 3. Results

The statistical analysis conducted on the aggregated dataset of *N* = 1038 observations revealed significant structural divergences in maneuver times, primarily governed by parking lot geometry and, to a decisive extent, by the spatial operational context. Notably for perception systems, these divergences generate distinct kinematic signatures rather than mere statistical noise.

The application of Generalized Linear Models (GLMs) allowed for the quantification of these differences, while the descriptive analysis presented in Table 1 provides a summary of key behavioral parameters for the monitored configurations.

In this new sensor-oriented framework, the 85th percentile value transcends its traditional role as a simple service capacity threshold. It becomes a fundamental operational “Ground Truth” metric, essential for calibrating the wait logic and dynamic timeouts in automated systems, teaching sensors to differentiate between natural human hesitations and actual static obstacles.

The results highlight that the variability in maneuver times reflects the complex physical interactions and kinematic challenges imposed by the various geometric configurations. This evidence has direct implications for the fine-tuning of exteroceptive sensors and the definition of prediction layers in Autonomous Vehicles (AVs). Furthermore, the analysis suggests that the temporal efficiency of a configuration is a dynamic variable, providing indispensable parameters for optimizing motion planning and object tracking algorithms in mixed-traffic environments.

### 3.1. Geometric Layouts and Kinematic Signatures for Sensor Calibration

The inferential investigation based on the Generalized Linear Model (GLM) delineated a clear performance hierarchy among the different parking lot types. The angled configuration (45°) unequivocally emerges as the Pareto-optimal behavioral baseline for training autonomous perception systems in high-density areas.

The data show that this geometry records the lowest mean entry time of the entire sample, at only 7.54 s, but the even more relevant figure for sensor calibration purposes is the minimal variance found (Std Dev = 8.90 s). This result indicates that oblique entry drastically reduces executive uncertainty: the 85th percentile impedance settles at 10.0 s, suggesting that the geometry itself minimizes the cognitive load required of the driver, facilitating vehicle alignment in a single fluid phase without the need for intermediate stops or trajectory corrections. This kinematic consistency translates into a highly predictable trajectory pattern, significantly optimizing the computational efficiency of object tracking modules. Furthermore, the reduced variance provides an extremely stable “Ground Truth” dataset, indicating that for this geometry, Autonomous Vehicle (AV) sensors can be calibrated to tighter temporal tolerances, given the low influence of individual differences in driving skills.

At the opposite pole in terms of predictive stability lies the parallel configuration (0°). Despite being geometrically simple and widespread in urban environments, it displays an intermediate but highly unstable performance, with a mean entry time of 12.68 s. The critical aspect for artificial perception is the significant increase in standard deviation (Std Dev = 13.68 s), which exceeds the mean value itself. This parameter reflects a strong dependence of the outcome on the individual driver’s skill and the availability of longitudinal space (gap size), making the parallel maneuver intrinsically stochastic. This high dispersion defines a “noisy” yet essential “Ground Truth” characteristic for sensor calibration: it demonstrates that, unlike other configurations, the prediction models (Motion Planning) of autonomous systems cannot rely on rigid deterministic thresholds. Instead, they must integrate a wider predictive uncertainty margin to prevent the system from erroneously classifying natural human hesitations, due to kinematic complexity and distance estimation, as anomalies or static obstacles

### 3.2. The Impact of Maneuvering Space: The Vertical Parking Paradox

**Figure 4:**
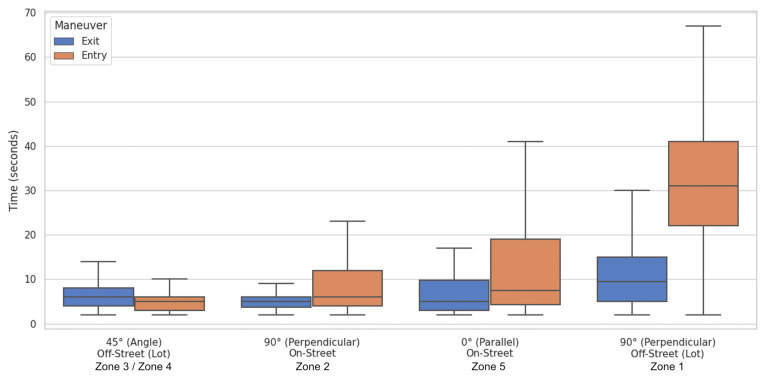
Comparative boxplot of maneuver time distributions.

**Figure 5:**
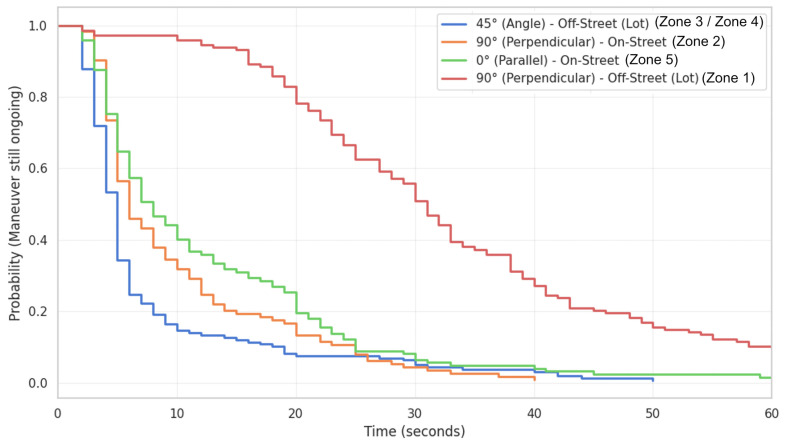
Impedance curves (Kaplan–Meier) for the entry maneuver.

The study’s most significant contribution lies in the analysis of the perpendicular configuration (90°), the efficiency of which proved not to be an intrinsic property of the parking lot geometry, but rather a variable strongly dependent on the available lateral maneuvering space. The comparison between the two monitored scenarios, illustrated in Figure 4 and Figure 5, reveals a performance dichotomy that we have defined as the “vertical parking paradox.”

In the “On-Street” context (Zone 2), where the orthogonal parking lot is accessible from a wide roadway acting as an extended virtual aisle, average entry times (9.84 s) are surprisingly comparable to those of angled parking (Figure 4). The availability of space allows drivers to optimize the entry maneuver by utilizing the opposite lane, assimilating the kinematics to that of an inclined parking lot. In this scenario, the wide turning radius permitted by the roadway compensates for the greater parking lot angle, reducing the need for corrective maneuvers and allowing for a more direct and fluid insertion. This demonstrates that, under ideal spatial conditions, the 90° configuration can be highly efficient and provides a “Ground Truth” baseline for validating optimal trajectories in automatic parking systems.

However, the scenario changes drastically in the “Off-Street Lot” context (Zone 1), characterized by a constrained 3.00 m aisle. In this confined environment, the average entry time triples, rising to 33.64 s. Even more critical from the perspective of system operability is the 85th percentile value, which reaches 50.1 s.

Survival Analysis confirms that temporal impedance in this scenario is not linear but dominated by multiple corrective maneuvers (saw-tooth maneuvers); the “tail” of the distribution extends well beyond 60 s, identifying these parking lots as the main bottlenecks of the university road system (Figure 5).

This data quantifies the operational cost of the geometric deficit: narrowing the aisle below the kinematic threshold of 5–6 m does not result in a linear degradation of performance, but rather a structural decay of entry capacity. The need to perform repeated reversals to align the vehicle not only exponentially increases maneuver times but also increases the risk of collisions and creates situations of prolonged blockage. From the perspective of artificial perception, the mapping of these iterations provides fundamental behavioral “Ground Truths”: sensors must be calibrated to recognize this fragmented kinematic pattern as a physiological maneuver induced by the geometric constraint, and not as a system anomaly or an unforeseen obstacle, thereby adapting timeout thresholds accordingly.

### 3.3. Functional Entry/Exit Asymmetry

A further piece of evidence transversal to all configurations, which emerges strongly from the inferential analysis, concerns the marked temporal asymmetry between maneuver phases. Exit operations systematically demonstrated a shorter duration compared to entry, refuting the simplifying assumption of symmetric impedance and providing precise temporal boundaries for trajectory prediction algorithms. The Generalized Linear Model (GLM) allowed for isolating and quantifying this phenomenon with statistical precision: as highlighted in Table 2, the coefficient associated with the variable Maneuver [Exit] assumes a negative value equal to −0.612, with extremely high statistical significance (*p*-value < 0.001). The structure of the model presented in Table 2 reflects the parsimonious specification derived from the methodological selection, where demographic and vehicular factors were omitted due to their verified lack of statistical influence. This outcome confirms that the temporal asymmetry and the infrastructural constraints act as the absolute dominant predictors of maneuver duration variance, thoroughly overshadowing individual driver characteristics.

In physical terms, given the logarithmic link function of the Gamma model, this coefficient indicates that the exit maneuver reduces the expected time by a factor of e^−0.612^ ≈ 0.54. This translates into an average time reduction of 46% (or, conversely, the exit requires approximately 54% of the entry time), all other conditions being equal.

**Figure 6:**
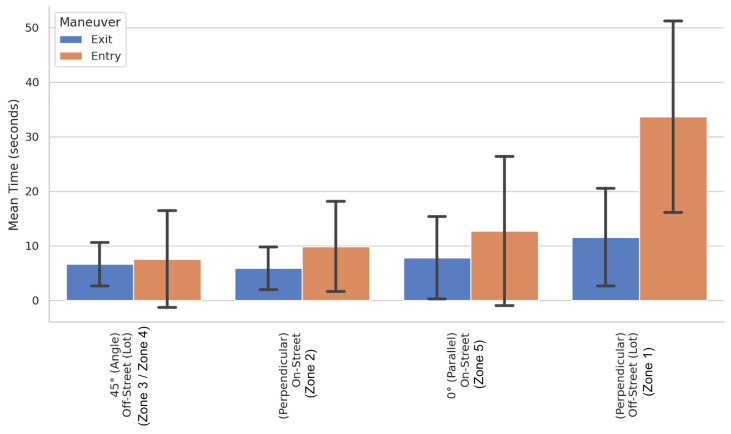
Comparison of mean maneuver times for Entry and Exit phases across all configurations.

This asymmetry is not uniform; rather, it is particularly pronounced in geometrically complex configurations. The most emblematic case is represented by the constrained perpendicular parking lot (Zone 1), illustrated in Figure 6, where the average time drops drastically from the 33.64 s required for entry to just 11.55 s for exit. This differential of over 20 s is explained by the distinct kinematic nature of the two operations: the entry phase acts as a process of precision positioning, requiring millimetric alignment and multiple corrections to center the vehicle within the parking lot’s lateral constraints; conversely, the exit phase functions as a process of disengagement, where the vehicle merely needs to gain sufficient space to merge into the traffic flow. Even in the presence of limited visibility, the exit geometry often allows the driver to clear the parking lot with a single continuous maneuver, bypassing the stop-and-go iterations typical of the entry phase.

The implications of this structural asymmetry are profound for the programming of interaction algorithms in autonomous systems. While treating maneuver time as a single constant leads to systemic errors in traditional capacity modeling, from an artificial perception perspective, this temporal dichotomy constitutes an indispensable behavioral “Ground Truth”.

Motion planning modules must adopt dynamic and differentiated timeout thresholds, assigning significantly wider Time-to-Clearance windows for vehicles during the entry phase compared to those exiting. Integrating these specific tolerances prevents the autonomous system from erroneously interpreting the structural slowness of the insertion maneuver as an anomalous roadway obstruction or a sensor failure.

## 4. Discussion

The interpretation of the experimental results offers a novel perspective on the relationship between infrastructural geometry, human behavior, and spatial constraints. Moving beyond the classical view that tends to treat maneuver time as a variable dependent almost exclusively on the parking lot inclination angle, data collected at the University Campus of Catania demonstrate that operational efficiency is the product of a complex and non-linear interaction between the geometric configuration of the parking lot, the type of maneuver, and the available lateral space (aisle width). These results are consistent with studies that have highlighted similar combined effects between parking lot/aisle dimensions and parking times [12,30,31,32]. In particular, this study extends the evidence already known for on-street parking regarding the effect of space length and maneuver type [12,30,31], introducing a specific quantitative “Ground Truth” often absent in Automated Valet Parking (AVP) literature, which tends to focus predominantly on algorithms in ideal or simulated geometries rather than on human baselines measured in the field [22,25,33,34,35,36].

Furthermore, to methodologically separate the influence of individual driver differences from infrastructural constraints, the study employed a Generalized Linear Model (GLM) framework. While the preliminary model explicitly controlled for demographic covariates (age, gender) and vehicle typologies, a rigorous stepwise model selection process revealed that their statistical significance was negligible compared to the explanatory power of the geometric layout and lateral spatial constraints. By excluding these variables to ensure statistical parsimony, the approach formally demonstrates that when lateral spatial constraints become severe, the physical impedance of the infrastructure statistically dominates subjective driving skills, producing kinematic signatures that are systematic and independent of the individual operator.

The inferential analysis confirms, in the first instance, the clear functional superiority of the angled parking configuration (45°), identified as the Pareto-optimal solution for speed (average of 7.54 s) and behavioral stability (σ = 8.90 s). This evidence links directly to literature demonstrating the greater spatial and operational efficiency of angled parking compared to vertical parking, especially in terms of single-movement maneuvers [35,36], in contrast to the increase in duration and perceived difficulty of 90° back-in maneuvers compared to smaller angles [32]. The reduced variance found at 45° provides AV developers with a “clean” dataset, essential for the training and validation of planners that prioritize fluid trajectories with minimal gear changes [22,25,33,34].

Diametrically opposed is the performance of the parallel configuration (0°). The high execution variability (σ = 13.68 s) detected here confirms the “non-holonomic” nature of the maneuver, aligning with research showing that times are extremely sensitive to the extra length available (where a 1 m increase can reduce times by up to 29–31%) [31] and follow non-normal and highly dispersed distributions for different entry/exit scenarios [12]. As the parallel scenario is particularly critical for planning and obstacle avoidance in dynamic environments [22,37,38], this intrinsic variability suggests that predictive models must integrate explicit uncertainty margins (e.g., in reward functions or decision-making modules) to manage the “noise” generated by human behavior [22,34].

However, the most critical contribution to defining a robust Human Baseline lies in the quantification of the “constraint cost” for the perpendicular configuration (90°), specifically the “vertical parking paradox”. The multiplicative factor of 4.46 on entry times in the presence of a reduced aisle (3.0 m) corroborates studies linking the reduction in useful dimensions to the exponential growth of duration and the number of forward–reverse movements [12,30,31,32]. The data clearly distinguish one-shot maneuvers from those characterized by multiple toing/froing, with severe impacts on spatial and operational efficiency [39]. Recognizing this fragmented “kinematic signature” is fundamental: motion planning models for tight spaces must explicitly include multiple forward/reverse segments [22,33] to avoid classifying such physiological maneuvers as anomalies.

A further novel element, with direct implications for Time-to-Clearance logic, is the parameterization of functional asymmetry. The finding that exiting is approximately 54% faster than entering is consistent with capacity studies showing that entry often occupies more time and is structurally dependent on geometry, while exiting is primarily constrained by gap acceptance and visibility [12,40,41], differentially influencing the capacity of intersections and signalized inlets [38]. For autonomous systems, this implies the need for “adaptive patience” that balances aggressiveness, comfort, and maneuver time through multi-objective optimization functions [22,25,33,34].

In conclusion, the definition of this reliable “Human Baseline” proves to be a prerequisite for the validation of Automated Valet Parking (AVP) algorithms [36].

The 85th percentile values emerging from the analysis are not simple descriptive statistics but represent operational “Ground Truth” thresholds indispensable for calibrating decision-making logic [22,25,34]. Without a comparison with these ground truth data, an autonomous vehicle would risk erroneously classifying a complex but physiological human maneuver as a road block (gridlock); the integration of these empirical tolerances into planning stacks (Motion Planning) is therefore essential to harmonize human–machine interaction and ensure the overall fluidity of the future road system.

## 5. Conclusions

The study provides a rigorous empirical quantification of parking maneuver durations in “Shared Space” contexts, establishing itself as an essential “Ground Truth” dataset to bridge the gap between deterministic traffic modeling and the stochastic requirements of autonomous driving systems. The analysis of 1038 naturalistic maneuvers does not merely delineate a hierarchy of geometric efficiency but establishes indispensable calibration parameters for the fine-tuning of perception and prediction algorithms (prediction layers).

Specifically, the angled parking configuration (45°) is identified as a stability benchmark for sensors, offering a behavioral signal with minimal impedance (7.54 s) and low variance (σ = 8.90 s), ideal for AV validation under laminar flow conditions. Conversely, the quantification of the vertical parking (90°) “paradox” provides a real-world “Edge Case” scenario: the data demonstrate that a lateral space deficit generates a complex and fragmented kinematic signature, with occupation times frequently exceeding 50 s (85th percentile). For computer vision systems, this data constitutes a critical “Ground Truth”: it trains the system not to classify repeated reversals as anomalies or static obstacles, but as physiological human adaptations to geometric constraints.

Furthermore, the parameterization of functional asymmetry, showing that exit operations structurally require approximately 54% less time than entry, offers an indispensable correction coefficient for timeout logic. The 85th percentile values isolated here are not simple statistics but become operational “Time-to-Clearance” thresholds: by integrating these probabilistic metrics, autonomous vehicles can modulate their algorithmic “patience,” distinguishing between a complex but legitimate human maneuver and a gridlock situation requiring active intervention.

Although the results are tied to the specific university context and the current reliance on offline frame-by-frame video extraction, they lay the foundation for a scalable library of behavioral “Ground Truths”. A natural progression of this research will involve framing the selection of the optimal parking geometry as a multi-objective optimization problem that balances these kinematic baselines with land consumption constraints. In this broader perspective, while the highly efficient 45° layout proves optimal for high-turnover scenarios prioritizing continuous flow, such as commercial hubs and freeway service areas, the 90° configuration retains a critical advantage in contexts demanding maximum spatial capacity, such as residential districts, despite its longer maneuvering times. Future perspectives will focus on fully automated trajectory acquisition via advanced computer vision algorithms in diverse geographical contexts, coupling kinematic metrics with spatial capacity models.

The ultimate goal is the development of comprehensive “Human–Machine Benchmarking” protocols to validate the safety and social acceptance of Automated Valet Parking (SAE Level 4) strategies in Smart Cities.

## Figures and Tables

**Table 1:** Descriptive statistics of maneuver times.

Context	Parking Angle	Maneuver	Count	Mean (s)	Std Dev (s)	85th %ile (s)
Zone 1	90°	Entry	147	33.64	17.55	50.1
		Exit	112	11.55	8.95	18
Zone 2	90°	Entry	113	9.84	8.27	20
		Exit	116	5.88	3.91	8
Zone 3	45°	Entry	157	7.54	8.9	10
Zone 4		Exit	165	6.61	3.96	10.4
Zone 5	0°	Entry	122	12.68	13.68	22
		Exit	106	7.77	7.56	14

**Table 2:** Generalized Linear Model (GLM) Regression Results.

Predictor	Coef.	Std. Err.	z	*p*-Value
Intercept	2.599	0.064	40.4	<0.001
Geometry [45° Angle]	−0.304	0.077	−3.95	<0.001
Geometry [90° Angle]	0.391	0.071	5.47	<0.001
Maneuver [Exit]	−0.612	0.055	−11.06	<0.001

## Data Availability

The original contributions presented in this study are included in the article. Further inquiries can be directed to the corresponding author.
